# Long-term use of denosumab and its association with skeletal-related events and osteonecrosis of the jaw

**DOI:** 10.1038/s41598-023-35308-z

**Published:** 2023-05-24

**Authors:** Pei-An Fu, Chin-Yao Shen, Shuen‑Ru Yang, Chun-Hui Lee, Hui-Wen Chen, Edward Chia-Cheng Lai, Wei-Pang Chung

**Affiliations:** 1 Department of Internal Medicine, National Cheng Kung University Hospital, College of Medicine, National Cheng Kung University, Tainan, Taiwan; 2 School of Pharmacy, Institute of Clinical Pharmacy and Pharmaceutical Sciences, College of Medicine, National Cheng Kung University, Tainan, Taiwan; 3 Department of Oncology, National Cheng Kung University Hospital, College of Medicine, National Cheng Kung University, No. 138, Sheng-Li Rd, Tainan, 704 Taiwan; 4 Department of Pathology, College of Medicine, National Cheng Kung University Hospital, National Cheng Kung University, Tainan, Taiwan; 5 Center for Applied Nanomedicine, National Cheng Kung University, Tainan, Taiwan

**Keywords:** Diseases, Medical research, Oncology

## Abstract

Denosumab, an inhibitor of receptor activator of nuclear factor kappa-B ligand, reduces skeletal-related events (SREs) and is approved for solid tumors with bone metastases. We studied long-term denosumab efficacy and safety because real-world data is scarce. This single-arm, single-center retrospective study included denosumab-treated breast cancer patients with bone metastases. Kaplan–Meier survival curves assessed exposure, SREs, osteonecrosis of the jaw (ONJ), and death. 132 patients were enrolled. The median denosumab exposure was 28.3 months (range 1.0–84.9). In the first year, 11.1% experienced SREs. This increased to 18.6% in the second, 21% in the third, and 35.1% in the fourth year and beyond. The median time to first on-study SRE has not been reached. 10 denosumab users (7.6%) developed ONJ. ONJ incidence was 0.9% in the first year, 6.2% in the second, 13.6% in the third, and 16.2% in subsequent years. The median time to first on-study ONJ has not been reached yet. Seven patients resumed denosumab after careful management of ONJ. Our data suggest that long-term treatment with denosumab may further prevent or postpone SREs at the cost of an increased risk of ONJ. The majority of patients who resumed denosumab did not experience a recurrence of ONJ.

## Introduction

Bone metastases are common in patients with metastatic breast cancer. Metastatic disease is found in up to 80% of individuals with metastatic breast cancer, potentially causing pain, hypercalcemia, and skeletal-related events (SREs)^1^. SREs include pathological fracture, bone irradiation, bone surgery, and spinal cord compression^1,2^, which exert significant, negative effects on patients' quality of life and survival^3,4^. Patients with bone metastases have increased expression of the receptor activator of nuclear factor kappa-B ligand (RANKL). Excessive RANKL-induced osteoclast activity causes bone resorption and bony destruction, leading to SREs. Denosumab, a fully human monoclonal antibody, inhibits osteoclast activity and reduces bone resorption, tumor-induced bone loss, and SREs by binding to human RANKL^1,5^.

Zoledronic acid is an intravenous bisphosphonate that is also a bone-targeted agent (BTA) that inhibits bone resorption; it is used to reduce SREs in bone metastasis^1,6^. Compared to zoledronic acid, denosumab delayed the time to the first on-study SRE by 18% in a phase III randomized control trial involving patients with breast cancer and bone metastasis [hazard ratio (HR) of 0.82)]^1^. Median time to first on-study SRE has not been reached in the denosumab arm. However, osteonecrosis of the jaw (ONJ) is an adverse event of special interest with denosumab. In this study, ONJ was infrequent in the denosumab group. Cumulative incidence was 0.8% at 1 year, 1.9% at 2 years, and 2.0% at 3 years. ONJ rates were not significantly different between the denosumab and zoledronic acid groups. Known risk factors for ONJ include dental extraction, poor oral hygiene, use of dental appliances, and anti-angiogenic therapy^1^.

Another phase III randomized control trial of patients with advanced cancers (excluding patients with breast or prostate cancer) also demonstrated the non-inferiority of denosumab to zoledronic acid^7^. The HR of delay time to the first on-study SRE was 0.84. For denosumab, the median time to first on-study SRE was 20.6 months; for zoledronic acid, it was 16.3 months. The two groups had similar progression-free and overall survival. Further, both groups demonstrated a similar incidence of ONJ. At one year, the cumulative incidence rates in the zoledronic acid and denosumab groups were 0.6% and 0.5%, respectively. After 2 years, 0.9% (zoledronic acid) and 1.1% (denosumab), and after 3 years, 1.3% (zoledronic acid) and 1.1% (denosumab). The clinical characteristics of ONJ were similar in both groups. Chemotherapy may be another risk factor for ONJ development. In this study, ~ 60% of patients with ONJ received chemotherapy.

Though SRE-prevention effects of denosumab have been demonstrated by several studies, the efficacy and side effects of extended denosumab therapy are less clear. In an open-label phase III trial involving 652 patients, extended denosumab treatment was administered to patients with metastatic breast or prostate cancers^8^. Among patients with breast cancer, the maximum exposure was up to 5 years, with a median duration of 19.1 months. In the denosumab group, the incidence of ONJ adjusted for years was 1.9%. Most cases were mild-to-moderate in severity and treated conservatively. The incidence rate increased with anti-resorptive therapy duration, and the median time to ONJ onset following treatment with denosumab was 20.6 months. As defined by complete mucosal coverage of exposed bone, ONJ resolved in 42% (35/83) of patients with breast cancer treated with denosumab or zoledronic acid. The median time to resolution in the denosumab group was 7.9 months.

To our knowledge, no study has reported real-world data on the use of denosumab beyond two years. We present the results of long-term denosumab use in patients with bone metastases treated in a tertiary medical hospital. Pre-education and ONJ prevention were provided to all patients. We examined the incidence of SREs with a focus on ONJ. We also documented the number of patients who safely resumed denosumab after fully recovering from ONJ.

## Patients and methods

### Study design

This was a single-arm, single-center retrospective cohort study that included patients with breast cancer who were administered denosumab to prevent SREs in bone metastases from December 2014 to November 2021 at a medical center in southern Taiwan. We implemented an as-treated approach. All patients received subcutaneous denosumab 120 mg Q4W, and were observed from their first administration of denosumab (the index date) to death, discontinuation, or the end of the study (8 November 2021). If denosumab exposure was paused for more than 2 months, it was defined as discontinuation. In this condition, observation for effectiveness outcome was stopped on the 30th day after the last administration. In addition, for safety outcomes, if the events happened within 90 days after discontinuation, it would be defined as denosumab-related event.

### Eligibility criteria

Eligible patients were ≥ 18 years old with histologically confirmed breast cancer. All patients demonstrated at least one radiographically (i.e., X-ray, computed tomography, or bone scintigraphy) or pathologically confirmed bone metastasis.

### Outcomes and covariates

The efficacy outcome was the first SRE after the index date. These included pathological fracture, bone irradiation, bone surgery, and spinal cord compression. The safety outcome was ONJ which was confirmed by pathological examination of tissue taken after surgical debridement. In addition, death would also be considered an outcome in this late-stage population once SREs and ONJ have been evaluated. Patients’ baseline characteristics included cancer subtypes, SREs history, menopausal status, and the Eastern Cooperative Oncology Group (ECOG) performance status scale. We also evaluated the use of bisphosphonates, vascular endothelial growth factor (VEGF) inhibitors, chemotherapy, and mandibular radiotherapy.

### Ethics

The study was approved by the Institutional Review Board of National Cheng Kung University of Taiwan (A-ER-111-259). We have gotten permission from the Institutional Review Board to waive informed consent. All studies were performed in accordance with the relevant guidelines and regulations.

### Statistical analysis

Categorical variables are numbers and percentages; continuous variables are represented as means and standard deviations. Exposure time, SREs, ONJ, and death were evaluated using Kaplan–Meier survival curves, and the median survival time was recorded. The cumulative incidence rate/probability of SREs and ONJ throughout the first 6 years were calculated by cumulative incidence function estimated by modeling the cause-specific hazard function. The incidence rates of SREs and ONJ before and after different cut-off timepoints were the ratio of the total outcome number to the total follow-up period^8^. We defined the cut-off timepoints as 12 months, 18 months, and 24 months. A swimmer plot was used to present patients’ follow-up after ONJ. SAS System 9.4 was used for all statistical analyses.

## Results

**Figure 1:**
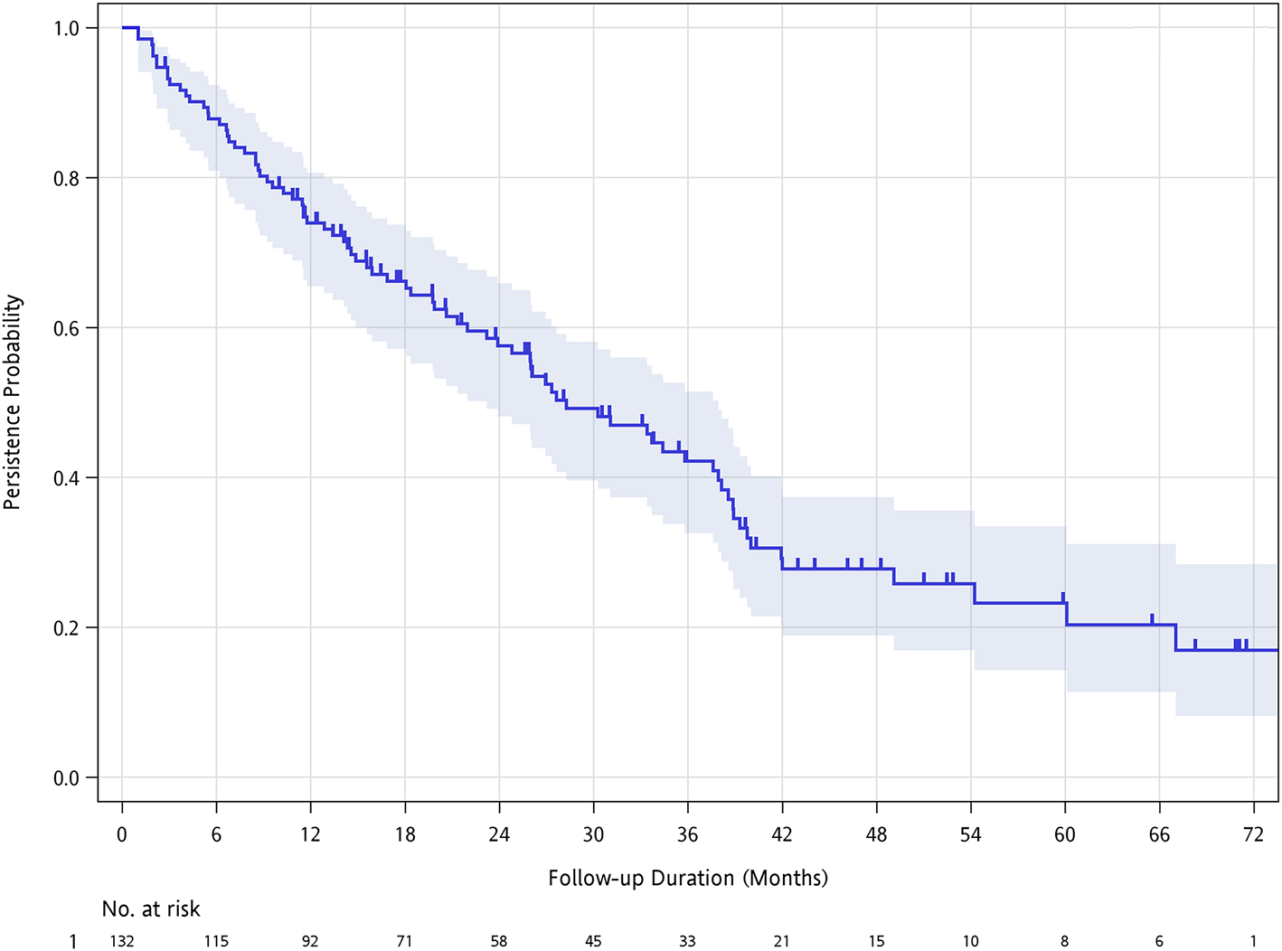
Exposure duration of denosumab in patients with metastatic breast cancer. Our patients' median length of denosumab exposure was 28.3 months; more than half continued denosumab for > 2 years.

Our study included 132 patients with breast cancer and bone metastasis (Table 1). The cohort’s median age was 55.1 years, and 66.4% of female patients were menopausal. Only one male patient was included, accounting for 0.8% of the cohort. The median denosumab exposure was 28.3 months (Fig. 1). The median duration of follow-up was 20.7 months (Supplementary Fig. 1). The follow-up and denosumab exposure ranged from 1.0 to 83.9 months. Nineteen patients (14.4%) experienced SREs before denosumab use. Other pre-denosumab medical interventions included: chemotherapy (n = 102; 77.3%), bisphosphonates (n = 14; 10.6%), bevacizumab: a humanized anti-VEGF monoclonal antibody (n = 2; 1.5%), and head and neck radiotherapy (n = 1; 0.8%).

**Table 1:** Patients’ baseline characteristics.

Age (years), median (SD)	55.1 (11.7)
Sex, n (%)	
Female	131 (99.2)
Male	1 (0.8)
Index year, n (%)	
2014	1 (0.8)
2015	13 (9.8)
2016	15 (11.4)
2017	22 (16.7)
2018	29 (22)
2019	20 (15.2)
2020	31 (23.5)
2021	1 (0.8)
Receptors status, n (%)	
ER/PR+ and HER2-	93 (70.5)
HER2+	26 (19.7)
Triple-negative	13 (9.8)
Menopausal status	87 (66.4)
ECOG status	
0–1	94 (71.2)
≥ 2	38 (28.8)
SREs before denosumab use	19 (14.4%)
Radiotherapy	19 (14.4%)
Bone surgery	2 (1.5%)
Prior use of bisphosphonate	14 (10.6%)
Prior exposure to chemotherapy	102 (77.3%)
Prior exposure to VEGF inhibitors	2 (1.5%)
Prior exposure to radiotherapy	1 (0.8%)

ER: estrogen receptor, PR: progesterone receptor, HER2: human epidermal growth factor receptor 2, ECOG: Eastern Cooperative Oncology Group, SREs: skeletal-related events, VEGF: vascular endothelial growth factor.

The causes of denosumab discontinuation were mortality or functional decline (n = 52; 39.3%), anticipated dental procedures or dental problems (n = 11; 8.3%) and ONJ (n = 10; 7.6%). Nine (6.8%) of patients were lost to follow-up. One patient discontinued denosumab because her breast cancer was considered cured. We identified only one patient who stopped denosumab due to dental problems and later developed SREs among the 73 patients who could be followed.

**Figure 2:**
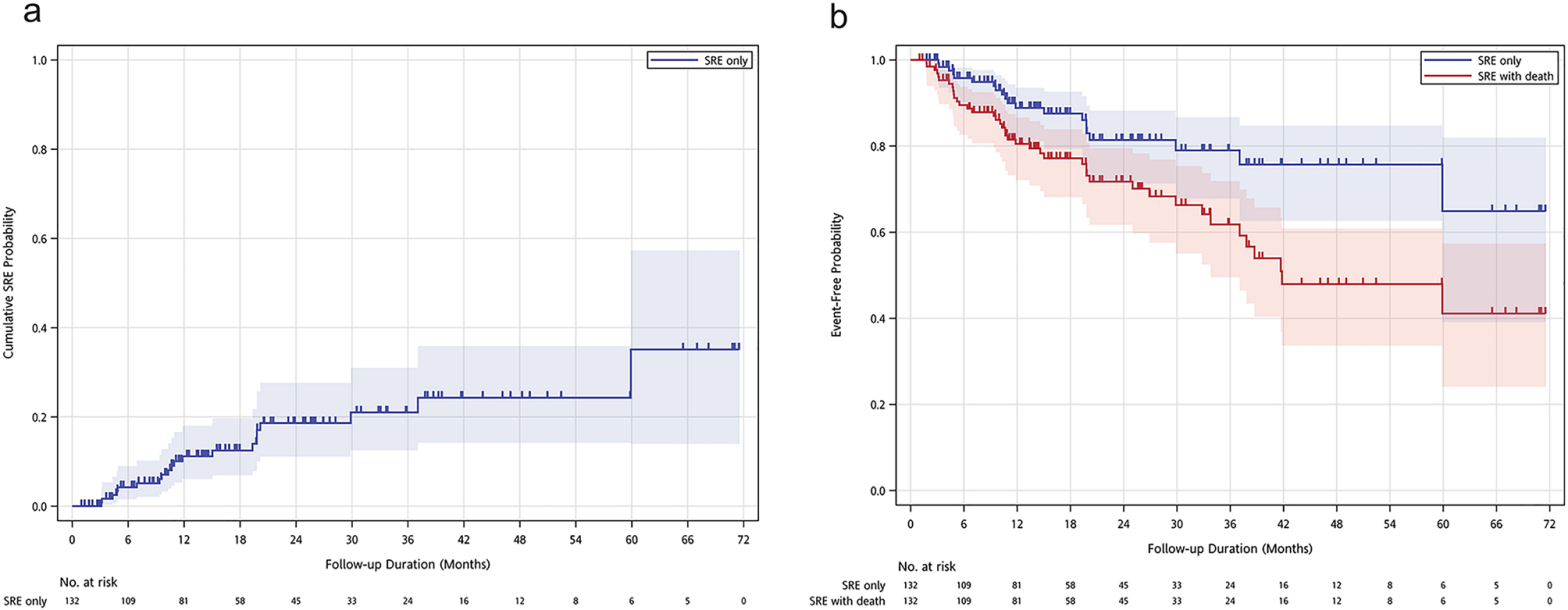
Time to the first skeletal-related event (SRE). (**a**) Cumulative incidence plot for SREs. (**b**) Kaplan–Meier survival curve for SREs and SREs with death. The median time from the first denosumab administration to the first on-study SRE has not been reached over 72 months of observation. Median SRE-free survival was 41.8 months.

**Figure 3:**
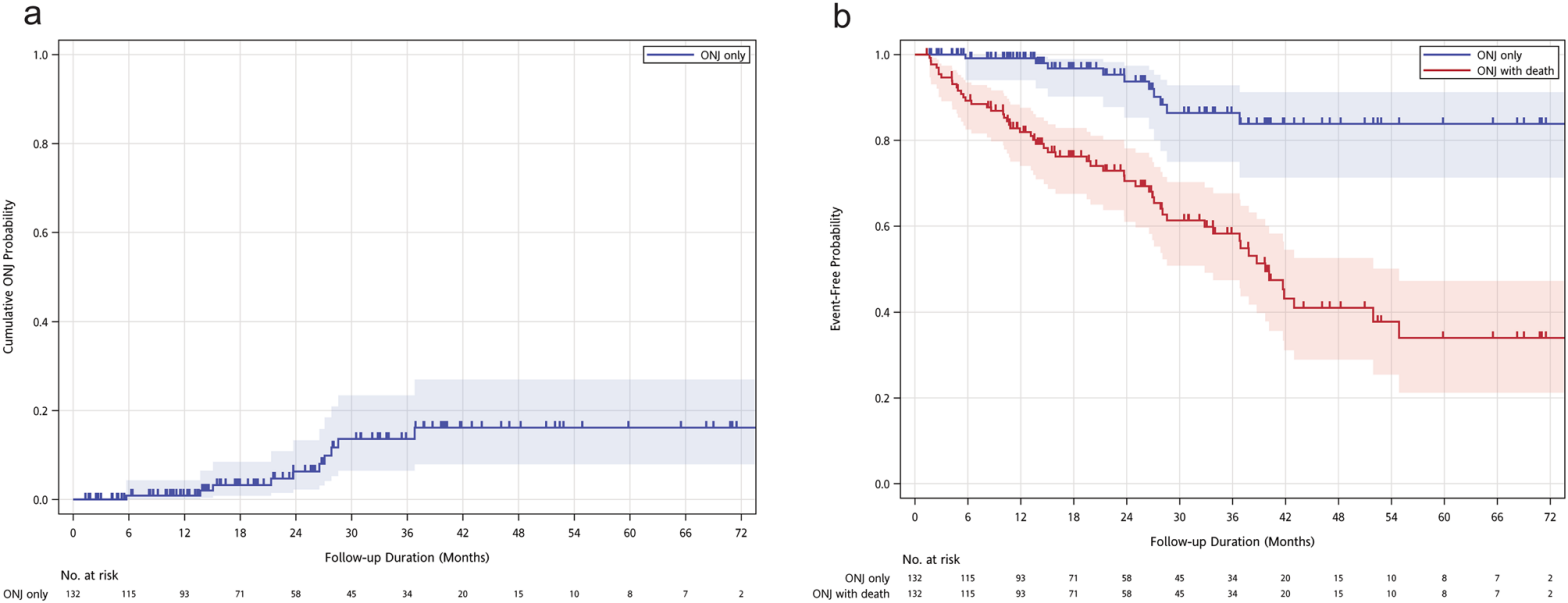
Time to the first osteonecrosis of the jaw (ONJ). (**a**) Cumulative incidence plot for ONJ. (**b**) Kaplan–Meier survival curve for ONJ and ONJ with death. The median time from the first on-study denosumab administration to the first ONJ has not been reached over 72 months of observation. Median ONJ-free survival was 39.7 months.

**Figure 4:**
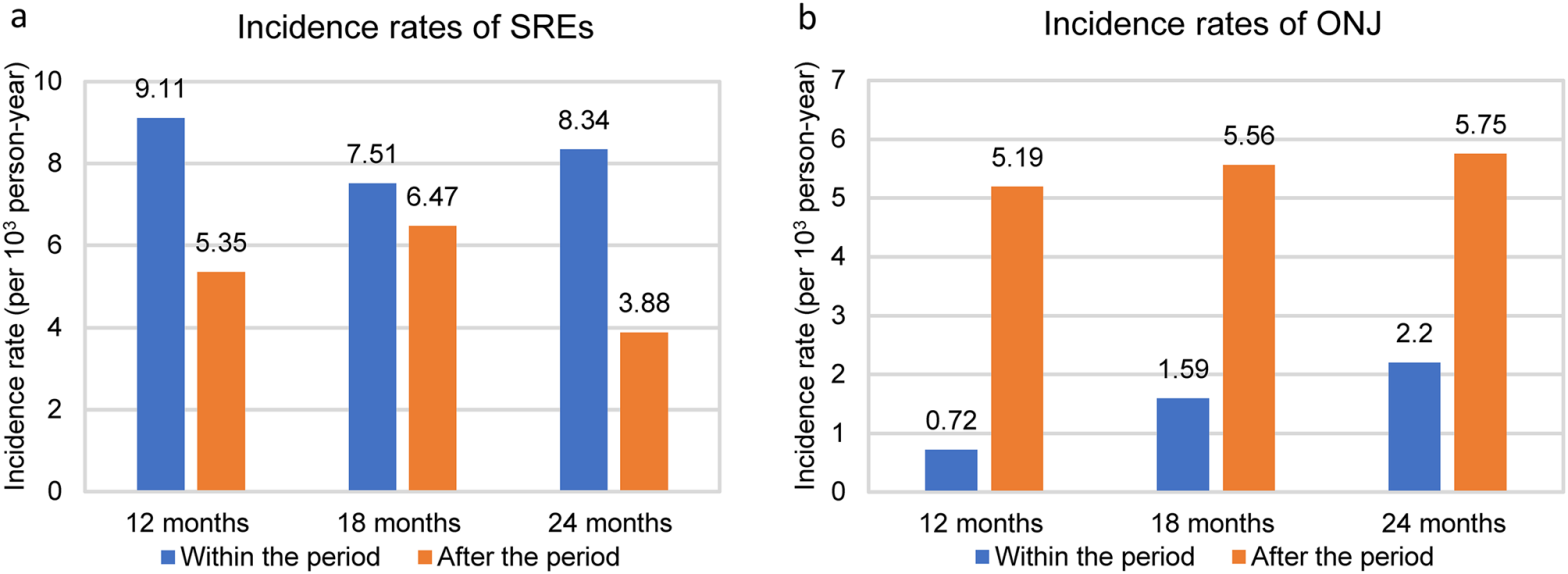
Incidence rates of skeletal-related events (SREs) and osteonecrosis of the jaw (ONJ) before and after three cut-off timepoints. (**a**) The incidence rates of SREs before and after three timepoints: 12 months, 18 months, and 24 months. (**b**) The incidence rates of ONJ before and after three timepoints.

**Figure 5:**
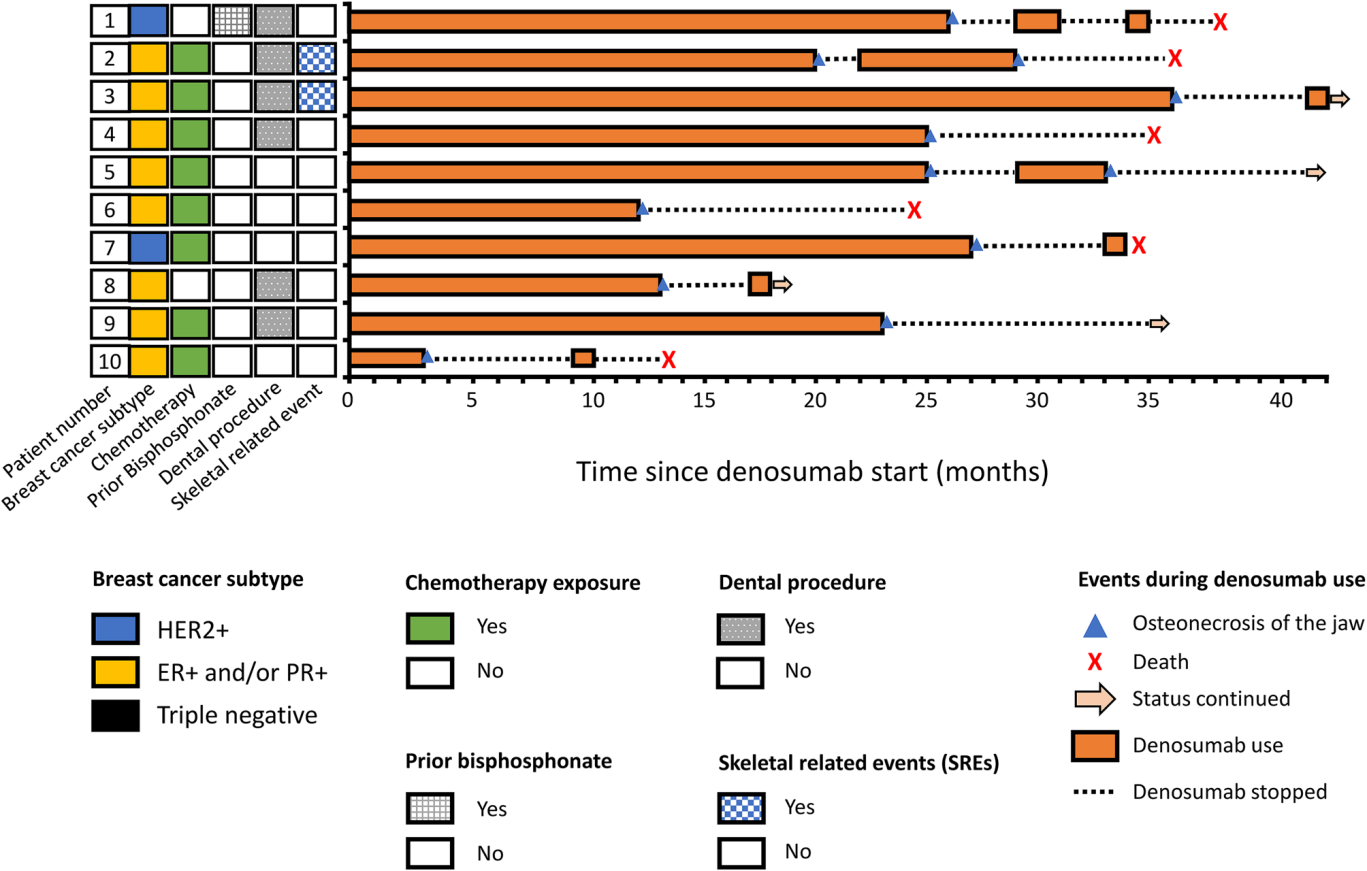
Timeline of denosumab usage for patients who experienced osteonecrosis of the jaw (ONJ). Using a swimmer plot, we demonstrated the course of denosumab use, the events, and the baseline characteristics of 10 patients with ONJ. Seven patients resumed denosumab after the management of ONJ. Eight patients had histories of chemotherapy. One patient was previously exposed to bisphosphonate. No patients had prior exposure to radiation to the head and neck or VEGF inhibitors. ER: estrogen receptor, PR: progesterone receptor, HER2: human epidermal growth factor receptor 2.

In our cohort, the cumulative SREs incidence was 11.1% in the first year, 18.6% in the second year, 21% in the third year, and 35.1% beyond the third year (Fig. 2a and Table 2). The median time to first on-study SRE has not been reached, and median SRE-free survival was 41.8 months (Fig. 2b). Two-year SRE-free survival rate was 77.2%, and five-year SRE-free survival rate was 41.1%. Of these patients with SREs, the events were all palliative radiotherapy for symptomatic relief, and none required orthopedic surgery. Ten patients (7.6%) developed ONJ while taking denosumab. The cumulative ONJ incidence probability was 0.9% in the first year, 6.2% in the second year, 13.6% in the third year, and 16.2% in the fourth year and beyond (Fig. 3a and Table 2). After denosumab use, the median time to first on-study ONJ has not been reached yet, and median ONJ-free survival was 39.7 months (Fig. 3b). Two-year ONJ-free survival rate was 70.5%, and five-year ONJ-free survival rate was 34%. The incidence rates of SREs after each cut-off timepoint were lower than before the respective time point (Fig. 4a). Nevertheless, the incidence rates of ONJ after each timepoint were higher than they were before the respective point (Fig. 4b). Among the 10 patients who developed ONJ, the time to onset ranged from 3 to 36 months (Fig. 5), and eight of them ever received chemotherapy. One patient had previous bisphosphonate exposure. None of the patients who developed ONJ received prior head and neck radiotherapy or VEGF inhibitors.

**Table 2:** Cumulative incidence probability of SREs and ONJ after denosumab use.

	1-year	2-year	3-year	4-year	5-year	6-year
SREs, n (%)	12 (11.1)	17 (18.6)	18 (21)	19 (24.3)	20 (35.1)	20(35.1)
ONJ, n (%)	1 (0.9)	5 (6)	9 (13.6)	10 (16.2)	10 (16.2)	10 (16.2)

SREs: skeletal-related events, ONJ: osteonecrosis of the jaw.

Seven of the 10 who experienced ONJ resumed denosumab after ONJ treatment, including surgical debridement (Fig. 5). The interval between two separate denosumab treatment courses was 2–6 months. The second course of denosumab was usually shorter than the first, and only two patients developed ONJ after the second course. The other three patients deferred a second course of denosumab due to performance status decline or disease progression. The other two patients resumed denosumab and continued therapy after the follow-up period ended.

## Discussion

Since the introduction of BTAs, including bisphosphonates and the RANKL inhibitor, the incidence of SREs in patients with cancer has dramatically decreased. Along with improved anti-cancer treatments, BTAs can potentially improve survival in patients with metastatic disease by preventing bone-related complications. Even though the optimal treatment duration with BTAs remains controversial, the American Society of Clinical Oncology recommends that BTAs should be used until the patient’s general performance status substantially declines^2^. The European Society for Medical Oncology also suggests that BTAs should continue indefinitely under normal circumstances^9^. Only one report has examined the use of BTAs—including denosumab—beyond 2 years of follow up in a real-world setting. This real-world cohort study estimated the risk of ONJ in a large population; however, the median denosumab exposure did not exceed one year^10^.

Judicious weighing of the pros and cons of prolonged denosumab use in patients with metastatic breast cancer is essential since survival in metastatic breast cancer patients continues to improve^11–13^. Additionally, denosumab demonstrates greater preference and persistence than other BTAs^14–18^. Our study included 132 patients with breast cancer who were administered denosumab to prevent bone metastasis-related SREs. Most of these patients demonstrated ER/PR-positive and HER2-negative subtype; 10.6% of patients received bisphosphonates before denosumab use. The maximum denosumab exposure was 83.9 months, and the median exposure was 28.3 months, longer than previous randomized control or real-world reports^1,8,10^.

Based on published studies, adverse reactions (especially ONJ) appear to increase with long-term BTA use^8,10^. Other recorded adverse reactions included nausea, arthralgia, hypocalcemia, and renal toxicity. Considering these potential adverse reactions, only ONJ led to denosumab discontinuation in our study. Other non-ONJ causes of denosumab discontinuation in our cohort included mortality or functional decline (39.3%) and anticipated dental procedures or dental problems (8.3%). A small percentage of patients (6.8%) were lost to follow-up. It has been warned that discontinuing denosumab in patients with osteoporosis will result in vertebral fractures^19^. In our cohort, only 1 in 73 patients experienced SREs after quitting denosumab due to an unavoidable cause.

Our study featured lower rates of SREs (11.1% in the first year, 18.6% in the second year, 21% in the third year, and 35.1% after fourth year) than other studies. In previous randomized trials, the SREs rates ranged 30%–40% in the first year^1,7^. Median time to first SRE in an integrated phase 3 randomized trial was 27.7 months^20^. In our study, the median time to first on-study SRE has not yet been reached. Two-year SRE-free survival rate was 71.7%, and five-year was 41.1%. Median duration of SRE-free survival was 41.8 months. According to previous pivotal studies, five-year survival rate was 52.3% among hormone receptor-positive, HER2-negative subtype^11^; and two-year survival rate on treatment was 42.1% in triple-negative subtype^12^, 80.5% in HER2-positive breast cancer^13^. Given that our analysis included patients with all subtypes of metastatic breast cancer, the SRE-free survival rates in our study may support the prolonged use of denosumab to prevent or postpone SREs and improve patients' quality of life in the end-stage. However, as mentioned before, the incidence of ONJ increased during long-term denosumab use^8,10,21^. Before beginning denosumab, patients at our facility will receive education from the breast cancer team members. They will have brochures detailing the importance of oral hygiene and the risk of developing ONJ during denosumab treatment. In addition, we advise our patients to report oral or dental events as soon as possible, to maintain good oral hygiene, and to consult with our team members prior to undergoing dental procedures. Once ONJ is suspected, an immediate referral to oral surgeon will be arranged. Even though efforts were made to prevent ONJ, 10 patients (7.6%) still developed the condition 3–36 months after initiating denosumab. The cumulative ONJ incidence probability was 0.9% in the first year, 6.0% in the second year, 13.6% in the third year, and 16.2% beyond the third year. Higher ONJ rates were reported in this study and may be the result of more long-term use of denosumab^8,10,21^. In studies involving shorter courses of denosumab use, the ONJ rate rarely exceeded 2%^1,7,22^. However, randomized trials with longer exposure to denosumab and extended follow-up periods consistently show a higher risk of ONJ. In one of these trials with extended follow-up, cumulative incidence of ONJ in the first 3 years was low^1^; but 6.3% (20/318) of patients still developed ONJ in the extension study, with a median denosumab exposure of 19.1 months^8^. Long-term denosumab use appears to be the main cause of ONJ, even given preventive efforts and increased awareness of this side effect. Additionally, real-world studies, by definition, include more complex patients and fewer controlled variables than clinical trials; thus, they may contribute to a higher ONJ rate in our study than in randomized trials. Because the cases of ONJ were limited, we did not classify the patients based on treatment duration to compare the outcomes of short-term and long-term treatments. However, our study provided a foundation for future studies to address this gap. Overall, the median time to first on-study ONJ after denosumab has not yet been reached, and five-year ONJ-free survival rate was 34%. Though ONJ appears to be an adverse event requiring more attention with prolonged denosumab use, it is still a manageable side effect which does not hinder denosumab application.

We thoroughly reviewed the risk factors for developing ONJ in these 10 patients. Eight patients received chemotherapy; most received eribulin or capecitabine when ONJ occurred. Taxanes and anti-metabolite agents were also administered to some patients. Neither facial bone radiotherapy nor VEGF inhibitors were administered. One of these 10 patients had prior exposure to bisphosphonate and this patient developed ONJ 26 months after shifting to denosumab. Some studies suggested a higher risk of ONJ and sooner ONJ development after transitioning from bisphosphonate to denosumab^23,24^. Our study did not reveal this transition to be a risk for developing ONJ. In our cohort, tooth extraction increased the risk of ONJ, similar to another study^22^. Six of the 10 patients in our study developed ONJ after tooth extraction. Four of these six patients received tooth extraction after a detailed discussion with our oral surgeons and knew the risk of ONJ. The extractions were unavoidable in these four patients due to infection or root instability; however, the other two patients underwent tooth extraction against medical advice. The causal relationship between denosumab and tooth extraction remains unclear. Denosumab could have aggravated a preexisting oral problem, or perhaps not. Seven of these 10 patients who experienced ONJ resumed denosumab use after fully recovering from oral debridement. A high rate of complete ONJ remission (70.5%) after treatment was also observed in another study^25^. Two patients developed recurrent ONJ after resuming denosumab. After the second occurrence of ONJ, resuming denosumab would no longer be considered. Five of seven patients tolerated the continued use of denosumab well without developing a second ONJ. However, the second course of denosumab was usually shorter than the first, mainly due to breast cancer progression. In these patients, denosumab was discontinued according to American Society of Clinical Oncology clinical practice guidelines^2^ since the patients’ general performance statuses significantly declined. Overall, resuming denosumab was generally safe after management and full recovery from ONJ. Long-term denosumab use was associated with excellent SRE-prevention outcomes. Although all patients were provided with comprehensive education and preventive strategies, the incidence of ONJ increased over time and was commonly associated with tooth extractions. Fortunately, some patients with ONJ continued denosumab treatment after receiving thorough medical care. In our cohort, prolonged use of denosumab could prevent patients from developing SREs at the expense of an increased incidence of ONJ, which were manageable in the majority of cases. These findings affirm existing recommendations that patients with metastatic bone disease should continue to use BTAs to prevent SREs. Recent research indicates that, among the various BTAs, denosumab demonstrates the most persistent benefits^14,15,18^. Providing patients with education, monitoring, and treatment for ONJ when needed enhances the benefits of denosumab.

## Data Availability

The datasets used and/or analyzed during the current study available from the corresponding author on reasonable request.

## Supplementary Figure

Supplementary Information 1.
