# Supplementary figures and images for: Long-term use of denosumab and its association with skeletal-related events and osteonecrosis of the jaw
Source: Sci Rep. 2023 May 24;13:8403. doi: 10.1038/s41598-023-35308-z (PMC10209178; doi:10.1038/s41598-023-35308-z)

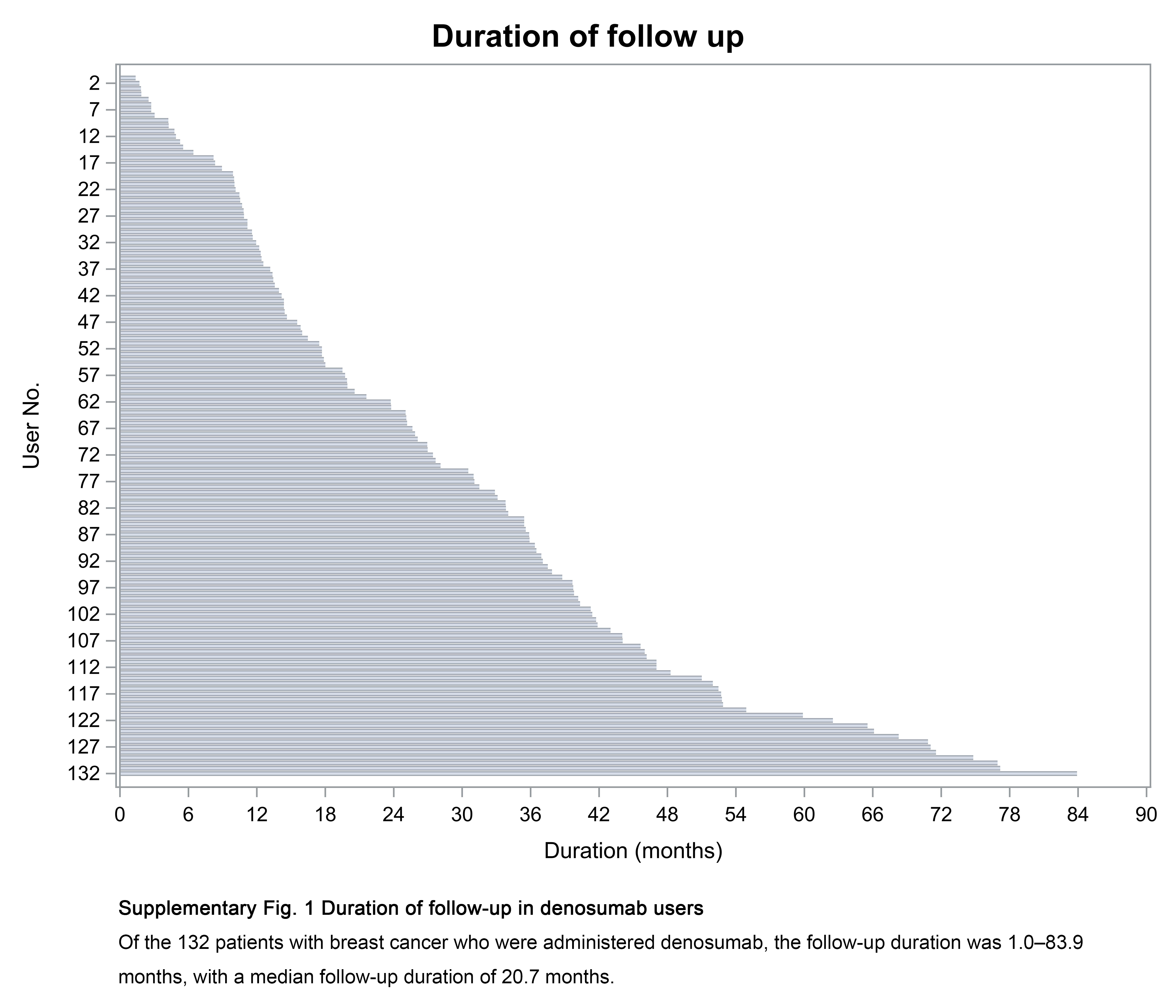

Supplement: Supplementary file 1 — Supplementary Information 1. [file 41598_2023_35308_MOESM1_ESM.tif]
